# Supplementary material for: A new duck genome reveals conserved and convergently evolved chromosome architectures of birds and mammals
Source: Gigascience. 2021 Jan 6;10(1):giaa142. doi: 10.1093/gigascience/giaa142 (PMC7787181; doi:10.1093/gigascience/giaa142)
Supplement: giaa142_Response_to_Reviewer_Comments_Original_Submission [file giaa142_response_to_reviewer_comments_original_submission.pdf]

Author's Response To Reviewer Comments

Close

Dear Hongling:

Thank you and the two reviewers' comments and suggestion on our manuscript. We have extensively revised our manuscript following reviewers' suggestion. We also highlighted the revised parts in our ms. Here are our point-to-point answers to reviewers' questions, and we hope you and the reviewers would find this new version of manuscript much improved to meet the requirement for publication on GigaScience.

Regards
--
Qi Zhou, PhD
Assistant Professor
Life Sciences Institute
Zhejiang University
Tel: +86-571-8898-1752
http://qizhoulab.net/

Referees' comments:
Reviewer #1: In this manuscript, Li and colleagues present a newly assembled genome of the Pekin duck, using multiple orthogonal sequencing and mapping technologies. The backbone of the assembly is generated with high-coverage PacBio long-read sequencing, followed by scaffolding with 10X-Genomics linked read sequencing, BioNano optical mapping and Hi-C chromatin interaction mapping. The authors analyze the duck genome by comparing it to chicken and emu, investigating chromosome sequence composition and report chromosome interaction domain patterns specific to the duck genome.
Overall, I enjoyed reading this manuscript and I think it is a solid contribution to the field of genomics. I have no major concerns regarding analyses or results, however there are quite a few minor issues regarding clarity (likely because of the quite dense manuscript). These are outlined as line-by-line comments below. I hope my comments are helpful and lead to an improvement of the manuscript.
A: We thank the reviewer for the positive and constructive comments.
Line 61: To my knowledge, there is no direct relationship between genome size and number of species in a given group of organisms. Seems odd to have this as an opening sentence.
A: Sorry for this misleading sentence, we revised it to 'Birds have the largest species number AND one of the smallest genome sizes among terrestrial vertebrates' to indicate the genome size and species number as two parallel traits without suggesting any connections.
Line 69: Since half of all bird species are passerines, stating that the majority of birds have the same karyotype is problematic, since the sampling is not phylogenetically independent. Hence a mechanistic relationship between an organism being a bird and having a certain karyotype cannot automatically assumed.
A: Here we wrote 'among the studied 800 bird species, the majority of them have a similar karyotype around 2n=80'. Therefore we are not assuming all the birds have the same karyotype.
Line 136: Why was a male duck used for the BioNano mapping (which is missing the W-chromosome)? (Same for the Hi-C prep below)
A: Thanks for pointing this out. The BioNano and Hi-C data were derived from the co-authors of this paper, who used the data for studying the domestication of Pekin duck, without particular interest into the sex chromosome evolution. We are at the moment producing the Hi-C data of a female duck, to improve the assembly of W chromosomes in our next version of duck genome.
Line 147: Above you write that Hi-C cannot be used to orient scaffolds, and in this sentence you state that there is a conflict in orientation between Hi-C and RH map. Is the conflict therefore within the Hi-C-based scaffold?
A: Thanks for pointing this out. Yes, 15 out of 69 conflicts are within the Hi-C based scaffold. And we have updated this information in the revised ms.
Figure 1 A and Line 136: BioNano doesn't produce 'read' data (as do sequencing technologies), but

rather maps.
A: We have changed the 'read' to 'maps' in Fig1a as suggested.
Line 161: "…, or alternative haplotype sequences not removed by purge haplotigs." I suppose 'purge haplotigs' is a step in the bioinformatic pipeline? Please clarify / rewrite.
A: Apologies for the confusion. We actually have purged the haplotigs. And we removed the phrase 'or alternative haplotype sequences not removed by purge haplotigs'.
Line 166: How were centromeres and telomeres annotated? Also, the total assembly size is 1,175 mb, corresponding to 83 % of the genome size estimation cited on line 129. While fewer contigs covering the assembly indeed mean reducing gaps, it may important to note that there are still ~200 Mb sequence missing from the assembly.
A: The centromeres and telomeres were annotated with their previously published consensus sequences. The details were presented in the 'Genome Annotation' section of Methods part. We have added the 200Mb sequence description at line 168 during this revision.
Table 1: Could you explain why the longest contig in the chicken assembly is more than twice as long as the longest in the ZJU1.0 duck assembly? Are there any particularly hard-to-assemble repeat regions at the breakpoints of these contigs?
A: Thanks for pointing this out. The longest contig of chicken assembly is on chr4, while the longest contig of duck ZJU1.0 is on chr3. So we can not compare them directly. We assume different sequencing technologies, coverage of linkage map and different repeat composition of the duck and chicken will affect the longest contig size. As chicken assembly is based on Sanger sequencing, while duck and zebra finch were based on PacBio sequencing. The size of the longest contigs of duck and zebra finch is similar, but shorter than chicken.
Line 198: Could you elaborate why gene density would be a factor increasing GC content on microchromosomes?
A: Because gene regions tend to have a higher GC content than non-coding regions, therefore gene density contributes to the different GC content on the microchromosomes, relative to the macrochromosomes.
Line 205: "assembled centromeres and telomeres" It would be interesting to know more about these structures (i.e. how long are the tandem repeat arrays?)
A: We now added the lengths of putative centromeres and telomeres in the revised text at line 212.
Line 233: Rather use 'sequence' instead of 'DNA'.
A: We have changed the 'DNA' to 'sequence' as suggested.
Line 366: "… because of some complex repeat sequences that accumulate at the boundary." Are the scaffold ends enriched for a certain type of DNA repeat?
A: The most abundant DNA repeat of the W scaffold ends is CR1-J2_Pass from LTR/ERVL.
Line 417: I would suggest to slightly alter the statement so that it becomes clear that the result reported is an observation rather than the outcome of an experiment. ".. revealed conserved mechanisms…" to me sounds like an experimentally proven causal relationship.
A: We agree, we now tuned down the statement as '...suggested conserved mechanisms..'.
Lines 426-428: This sentence is unclear to me; why is the gene conversion mediated by palindromes "despite" the fact that gene copies have become pseudogenes?
A: We have revised the sentence as 'despite the repair mechanism mediated by gene conversions between gene copies within the palindromes' to clarify it.
Lines 435-438: There are three 'may' in two sentences. Please re-write for clarity.
A: We have revised the two sentences.
Lines 520-523: Ordering W scaffolds based on their collinearity with the Z-chromosome excludes any rearrangements between Z and W chromosome per se. This is problematic in my opinion.
A: We now added a statement following that sentence clarifying that there are probably rearrangements between the chrZ and chrW, and our chrW sequences do not reflect their actual order in the genome. As we mentioned above, we are producing the Hi-C data of a female duck and trying to improve the assembly of W chromosomes in our next version of duck genome.

Reviewer #2: This manuscript presents a new duck genome assembly which is greatly improved over past duck genome assemblies. The manuscript presents detailed analysis of the genomic structure of the duck chromosome Z. The genome assembly should be a valuable resource for the bird genomics community, and the analyses of sex chromosome were interesting and thorough.
I think that Data Description section is much more detailed than the journal describes (below taken from the Instructions to Authors:
"A statement providing background and purpose for collection of these data should be presented for readers without specialist knowledge in that area. A brief description of the protocol for data collection, data curation and quality control, as well as potential uses should be included, as well as outlining how the data can be accessed if it is not deposited in our repository."

I think the Data Description needs to be greatly reduced from the current 2.5 pages to one or 1.5 pages. I would suggest moving of the discussion of the error correction and improvements in genome completeness and annotation to the analyses and discussion sections of the paper.
A: We have now put the comparison to the previous assembly, and genome annotation into the analysis part, as suggested by the reviewer. Now the Data Description is about 1 page.
There are other sections with need to be reorganized for clarity, along with minor revisions throughout, which I have detailed in the attached comments.
Detailed review of "A new duck genome reveals conserved and convergently evolved chromosome architectures of birds and mammals"

Line 49-52: Replace "Parallel" with "Similar", "a sequence divergence pattern" with "a pattern of sequence divergence".
A: We have replaced the word and phrase as suggested.
Line 61: replace "one of the smallest genome sizes" with "some of the smallest genomes"
A: We have replaced the phrase as suggested.
Line 62: Rewrite this sentence. It gives the impression that the tremendous phenotypic diversity of birds emerged "since the era of cytogenetics".

A: Thanks for your suggestion. We have moved "since the era of cytogenetics" to the front part of the sentence.

Line 89: suggest replacing "retard" with "limit"

A: We have replaced "retard" with "limit" in the manuscript.

Line 112: suggest rewriting this sentence to read, "with all the cutting-edge technologies mentioned above. We corroborated our reference genome through comparisons to previously published …"

A: We have revised the sentence as suggested.

Line 113: don't need to capitalize "Fluorescence"

A: We have replaced the word as suggested..

Line 115: suggest removing "(chicken and turkey etc.)".

A: We have removed the phrase as suggested.

Line 119: It isn't clear what "they" refers to here. Is it the duck sex chromosomes, the duck, emu and chicken sex chromosomes, all three genomes together?
Line 119-121: The chronological order referred to in this sentence isn't clear. You previously referred to the divergence time of Anseriformes from Galliformes but didn't provide the divergence time of emu. If emu isn't part of that chronology, then it isn't clear because you just stated in the previous sentence that duck sex chromosomes are intermediate between chicken and emu.

A: We have revised the sentence here to "The gradient of sex chromosome divergence levels exhibited by the three bird species together.." to clarify that we are referring to all three species together.

Line 133: replaces "sequences" with "bases"

A: We have replaced the word as suggested.

Line 134-137:
1) Replace "-fold" with "-X genome coverage",

A: We have replaced the word as suggested.

2) The Hi-C and Bionano data was from a male duck, the PacBio and 10X data from a female duck. The relatedness or not of the two sequenced individuals should be included.

A: These data were derived from different individuals, regardless male or female, from the same inbred duck strain. The detailed relatedness of sequenced individuals can be seen in Supplementary Table S1.

3) The read N50 for the PacBio reads given in the text is 14.3 kb, but Suppl. Fig. S1 has a read N50 of 15,333 bp. The caption for Supplementary Figure S1 states that the figure is the length distribution of subreads from one SMRT cell, but there must have been multiple SMRT cells used to get 143-X genome coverage. The number of SMRT cells used should be provided either in the main text of the paper or in the caption of Supplementary Figure S1. Something like "Length distribution from one representative SMRT cell out of X SMRT cells" in the figure title for example.

A: We presented in the main text the N50 for all the PacBio data which is 14.3kb, while Supp. Fig.1 presented an example of one SMRT cell, whose N50 is 15.3kb. We now included the information of SMRT cell numbers in the main text, and also changed the title of Supplementary Figure S1 accordingly.

Line 137: Was the illumina data generated from the same male individual as was used for the Hi-C and Bionano data? If not, how was he related or not to the other ducks used?

A: We added "of the same duck strain" after "two different male individuals" to clarify. These data were derived from different individuals, regardless male or female, from the same inbred duck strain. The detailed information of sequenced individuals can be found in Supplementary table S1.

Line 139: need citation, preferably URL or bioproject number at the NCBI’s SRA, for the "previously published female reads"

A: The female illumina reads were sequenced by our co-authors and have been uploaded at the NCBI's SRA. (SRR11906239-SRR11906245, SRR11906251, SRR11906258-SRR11906263 from Bioproject PRAJNA 636121)

Line 141-142: Should refer to Table 1 here since that is where this data is presented.

A: We referred to Table 1 here as suggested.

Line 149: what software was used for correcting the orientation errors?

A: We wrote python scripts to correct the orientation errors. The scripts are shared in Github (https://github.com/ZhouQiLab/DuckGenome).

Line 154: I think you need to present some data (a supplementary figure) to show that the final polished assembly is consistent with the FISH linkage map.

A: We have added the Supplementary figure S2 to show the final polished assembly is consistent with the FISH linkage map.

Line 155-156: I think this sentence is interesting, but doesn’t belong in this section of the paper. It should go where the "see below" indicates.

A: We mainly put this sentence here to indicate that our assembly quality is high, without chimeric assembly of Z- and W-linked sequences into one sequence, as indicated by the coverage results mentioned here.

Line 160-162: Should state what part of the pipeline "purge haplotigs" is in.
A: Apologies for the confusion. We actually have purged the haplotigs. And we removed the phrase 'or alternative haplotype sequences not removed by purge haplotigs'.
Line 162: Change "macrochromosomes" to "assembled macrochromosomes".

A: We have replaced the word as suggested.

Line 164: Data should be presented to support the assembly of the microchromosomes.

A: We referred the data to Figure 2a here during the revision.

Line 176: Should clarify "evolutionarily young". Young relative to what?

A: Here the age of repeats was measured by their divergence level from the consensus sequences or

whether they inserted into another repeat. We now clarified them in the text as ‘young repeat relative to repeats of the same family’.

Line 184: Replace "recovered" with "identified" or "annotated", "from" with "in", "of which" with "including".

A: We have replaced the words and the phrase as suggested.

Analyses

Line 193: replace "micro-chromosome" with "microchromosome"

A: We have replaced the word as suggested.

Line 201: rewrite to "genes on chrZ are expressed at twice the level in males versus females"

A: We have rewritten the sentence as suggested.

Line 202-204: If the expression of genes on chrZ is double in males versus females, doesn’t that mean that chrZ exhibits dosage compensation in females? Maybe need to rewrite this sentence and the previous sentence to clarify.

A: Dosage compensation evolved to balance the expression imbalance between the autosomes and sex chromosomes in the heterogametic sex, which further results in an equal expression between male and female on the sex chromosomes. Therefore, a 2-fold difference of expression level on the chrZ between sexes indicated a lack of dosage compensation in female birds.

Line 225: delete "on"

A: We have deleted the word as suggested.

Line 241: replace "have not found" with "did not find"

A: We have replaced the phrase as suggested.

Line 247: remove "identified"

A: We have removed the word as suggested.

Line 251: Some more information about the location of this gene, gene annotation information or at least chromosome location, should be provided.

A: Thanks for your suggestion. The RNF135 gene is located on duck chr19 and has been added in the manuscript.

Line 313: suggest replacing "some tissue" with "certain tissues".

A: We have replaced the phrase as suggested.

Line 320-321: Rewrite to clarify that "their" is the sex chromosomes of Pekin duck, not Pekin duck.

A: We have rewritten the sentences as suggested.

Line 329: remove "of"

A: We have removed the word as suggested.

Line 328-350: this entire paragraph needs to be reorganized for clarity and should be broken into at least two paragraphs. One should summarize the assembly status of chrZ in the new assembly. How much sequence could be anchored into the largest scaffold? What percentage of the expected chromosome length is that? The second paragraph should be about the PAR. The next paragraph should be about the large tandem arrays. Next the duck chrW should be its own paragraph.

A: We have now divided this paragraph into three paragraphs as suggested by the reviewer. We also included information of the numbers of Z-linked scaffolds. Because we do not have an estimated or expected size of duck chrZ, we compared it to the chicken chrZ.

Line 353: "reshuffling" not "reshufflings"

A: We have replaced the word as suggested.

Line 365: "did not find" not "have not found"
A: We have replaced the phrase as suggested.

Line 374: "remove "the"

A: We have removed the word as suggested.

Line 378: "concentrated in those families" not "at those families"

A: We have rewritten the sentences as suggested.

Line 431: "the early stage of avian sex chromosome evolution". This is inaccurate. The emu sex chromosomes have been evolving just as long as the chicken and duck sex chromosomes. The emu sex chromosomes are just not as differentiated.

A: We have revised the sentence as '...emu chrW.., which evolves much slower than chrWs of chicken and duck'.

Line 433: rewrite to "sex-linked palindromes are a feature of strongly differentiated sex chromosomes which have accumulated abundant TEs", see above suggestion.

A: We have rewritten the sentences as suggested.

Line 445: SMRT cells weren't generated, they were sequenced.

A: We have rewritten the sentences as suggested.

Line 614,615: replace "micro-chromosome" with "microchromosome"

A: We have replaced the word as suggested.

Line 641: Evolutionary, not Evolution

A: We have rewritten the sentences as suggested.

Line 655: Table 1 and the Figure Legends should be with the figures, not with the works cited list between Table 1 and the figures.
A: Thanks for your suggestion. We have moved the figure legends with the figures.

Figure 1: This figure indicates many softwares that weren't cited previously but should be cited.
A: We have now cited the software in the 'Genome Assembly' section of Methods part.

Supplementary Figure S1: The read N50 for the PacBio reads given in the text is 14.3 kb, but Suppl. Fig. S1 has a read N50 of 15,333 bp. The caption for Supplementary Figure S1 states that the figure is the length distribution of subreads from one SMRT cell, but there must have been multiple SMRT cells used to get 143-X genome coverage. The number of SMRT cells used should be provided either in the main text of the paper or in the caption of Supplementary Figure S1. Something like "Length distribution from one representative SMRT cell out of X SMRT cells" in the figure title for example. Could also remove this figure entirely.

A: The difference of the N50 number is because in the main text we showed the N50 length for all the SMRT cells, while in the Supplementary Fig. 1, we showed N50 length for one SMRT cell as an example, Now we have changed the title as suggested by the reviewer.

Supplementary Figure S2: Suggest changing figure title to "A representative case of assembly error correction".

A: We have changed the figure title as suggested.

Supplementary Figure S14: Should cite ggplot2 package here.

A: We have cited the ggplot2 package as suggested.

Supplementary Figure S23: Should indicate how the strata were identified. Just a brief indication to help the reader find it in the methods.

A: We have added the indication in the figure legend.

Supplementary Table S1: Were the 10X reads paired or not? There should be an additional column in this table indicating which individuals were used for sequencing for each technology. For example, was the same female used for PacBio and 10X sequencing? Were the Illumina reads, male and female, paired-end? I think the data category for the "Read Length/N50" column should be included in each cell. Otherwise, a reasonable reader could think that the Bionano data has a read length of 325,300 basepairs (which is actually map data, not sequence reads); I suggest changing the PacBio and Bionano columns to include "(N50)" next to the basepair unit.
A: We have added another two columns to indicate the information of "paired or not" and "sequencing individuals". Also, we changed the "Read Length/N50" to "Read/Map length" and add the N50 values in each cell.

Supplementary Table S2: This table is very helpful to show the various parameters used in each assembly, but it also raises a couple of questions about the PacBio data. Suppl. Figure S1 showed the read length distribution from an RSII SMRT cell, but this table indicates that both sequel and RSII data was generated. It should be stated in the main text how many SMRT cells were used, and probably in Supplementary Table S1 how much sequence was generated with each type of instrument.

A: We have added the information of SMRT cell numbers in the main text, and also added the sequence information of each type of instrument in Supplementary Table S1.

Line 142 – 144: Need software used for error-correction, and orientation and "connected". I think "connected" should be replaced with "scaffolded".

A: Thanks for your suggestion. We used our own scripts to correct the orientation errors. The scripts have been uploaded in Github (https://github.com/ZhouQiLab/DuckGenome). We also replaced the word as suggested.

Line 145-47: What software was used for incorporating the linkage map? Suggest rewriting to: "… we incorprorated an RH linkage map[32], which reduced …"

A: First we aligned the RH linkage map to the scaffold-level duck assembly with nucmer software, to determine the scaffold order within the chromosome. Then we wrote our own python script to link the scaffolds into chromosomes. The script has been uploaded in Github (https://github.com/ZhouQiLab/DuckGenome). We also revised the text as suggested.

Supplementary Table S3: What is the "ZJU1.0" column indicating here? Is it the length of the chromosome in the final polished assembly? If so, suggest changing the column name to be "chr. length" and changing the table name to be "Chromosome anchoring in ZJU1.0 assembly".

A: We have changed "ZJU1.0" to "chr.length" and the table name to "Chromosome anchoring in ZJU1.0 assembly" as suggested.

Close
