# Supplementary material for: A new duck genome reveals conserved and convergently evolved chromosome architectures of birds and mammals
Source: Gigascience. 2021 Jan 6;10(1):giaa142. doi: 10.1093/gigascience/giaa142 (PMC7787181; doi:10.1093/gigascience/giaa142)
Supplement: giaa142_Reviewer_1_Report_Original_Submission — Matthias Weissensteiner -- 10/6/2020 Reviewed [file giaa142_reviewer_1_report_original_submission.pdf]

Reviewer Report

Title: A new duck genome reveals conserved and convergently evolved chromosome architectures of birds and mammals

Version: Original Submission **Date:** 10/6/2020

Reviewer name: Matthias Weissensteiner

Reviewer Comments to Author:

In this manuscript, Li and colleagues present a newly assembled genome of the Pekin duck, using multiple orthogonal sequencing and mapping technologies. The backbone of the assembly is generated with high-coverage PacBio long-read sequencing, , followed by scaffolding with 10X-Genomics linked read sequencing, BioNano optical mapping and Hi-C chromatin interaction mapping. The authors analyze the duck genome by comparing it to chicken and emu, investigating chromosome sequence composition and report chromosome interaction domain patterns specific to the duck genome.
Overall, I enjoyed reading this manuscript and I think it is a solid contribution to the field of genomics. I have no major concerns regarding analyses or results, however there are quite a few minor issues regarding clarity (likely because of the quite dense manuscript). These are outlined as line-by-line comments below. I hope my comments are helpful and lead to an improvement of the manuscript.
Line 61: To my knowledge, there is no direct relationship between genome size and number of species in a given group of organisms. Seems odd to have this as an opening sentence.
Line 69: Since half of all bird species are passerines, stating that the majority of birds have the same karyotype is problematic, since the sampling is not phylogenetically independent. Hence a mechanistic relationship between an organism being a bird and having a certain karyotype cannot automatically assumed.
Line 136: Why was a male duck used for the BioNano mapping (which is missing the W-chromosme)? (Same for the Hi-C prep below)
Line 147: Above you write that Hi-C cannot be used to orient scaffolds, and in this sentence you state that there is a conflict in orientation between Hi-C and RH map. Is the conflict therefore within the Hi-C-based scaffold?
Figure 1 A and Line 136: BioNano doesn't produce 'read' data (as do sequencing technologies), but rather maps.
Line 161: "…, or alternative haplotype sequences not removed by purge haplotigs." I suppose 'purge haplotigs' is a step in the bioinformatic pipeline? Please clarify / rewrite.
Line 166: How were centromeres and telomeres annotated? Also, the total assembly size is 1,175 mb, corresponding to 83 % of the genome size estimation cited on line 129. While fewer contigs covering the assembly indeed mean reducing gaps, it may important to note that there are still ~200 Mb sequence missing from the assembly.
Table 1: Could you explain why the longest contig in the chicken assembly is more than twice as long as the longest in the ZJU1.0 duck assembly? Are there any particularly hard-to-assemble repeat regions at the breakpoints of these contigs?

Line 198: Could you elaborate why gene density would be a factor increasing GC content on microchromosomes?
Line 205: "assembled centromeres and telomeres" It would be interesting to know more about these structures (i.e. how long are the tandem repeat arrays?)
Line 233: Rather use 'sequence' instead of 'DNA'
Line 366: "… because of some complex repeat sequences that accumulate at the boundary." Are the scaffold ends enriched for a certain type of DNA repeat?
Line 417: I would suggest to slightly alter the statement so that it becomes clear that the result reported is an observation rather than the outcome of an experiment. ".. revealed conserved mechanisms…" to me sounds like an experimentally proven causal relationship.
Lines 426-428: This sentence is unclear to me; why is the gene conversion mediated by palindromes "despite" the fact that gene copies have become pseudogenes?
Lines 435-438: There are three 'may' in two sentences. Please re-write for clarity.
Lines 520-523: Ordering W scaffolds based on their collinearity with the Z-chromosome excludes any rearrangements between Z and W chromosome per se. This is problematic in my opinion.

Methods

Are the methods appropriate to the aims of the study, are they well described, and are necessary controls included? Choose an item.

Conclusions

Are the conclusions adequately supported by the data shown? Choose an item.

Reporting Standards

Does the manuscript adhere to the journal's guidelines on minimum standards of reporting? Choose an item.

Choose an item.

Statistics

Are you able to assess all statistics in the manuscript, including the appropriateness of statistical tests used? Choose an item.

Quality of Written English

Please indicate the quality of language in the manuscript: Choose an item.

Declaration of Competing Interests

Please complete a declaration of competing interests, considering the following questions:

- Have you in the past five years received reimbursements, fees, funding, or salary from an organisation that may in any way gain or lose financially from the publication of this manuscript, either now or in the future?
- Do you hold any stocks or shares in an organisation that may in any way gain or lose financially from the publication of this manuscript, either now or in the future?
- Do you hold or are you currently applying for any patents relating to the content of the manuscript?
- Have you received reimbursements, fees, funding, or salary from an organization that holds or has applied for patents relating to the content of the manuscript?
- Do you have any other financial competing interests?
- Do you have any non-financial competing interests in relation to this paper?

If you can answer no to all of the above, write 'I declare that I have no competing interests' below. If your reply is yes to any, please give details below.

I declare that I have no competing interests.

I agree to the open peer review policy of the journal. I understand that my name will be included on my report to the authors and, if the manuscript is accepted for publication, my named report including any attachments I upload will be posted on the website along with the authors' responses. I agree for my report to be made available under an Open Access Creative Commons CC-BY license (http://creativecommons.org/licenses/by/4.0/). I understand that any comments which I do not wish to be included in my named report can be included as confidential comments to the editors, which will not be published.

Choose an item.

To further support our reviewers, we have joined with Publons, where you can gain additional credit to further highlight your hard work (see: https://publons.com/journal/530/gigascience). On publication of this paper, your review will be automatically added to Publons, you can then choose whether or not to claim your Publons credit. I understand this statement.

Yes Choose an item.
