# Supplementary material for: A new duck genome reveals conserved and convergently evolved chromosome architectures of birds and mammals
Source: Gigascience. 2021 Jan 6;10(1):giaa142. doi: 10.1093/gigascience/giaa142 (PMC7787181; doi:10.1093/gigascience/giaa142)
Supplement: giaa142_Supplemental_Files [file giaa142_supplemental_files.zip › Round 1-Li et al Duck genome review comments Daniel Ence.docx]

Daniel Ence Detailed review of “A new duck genome reveals conserved and convergently evolved chromosome architectures of birds and mammals”

Line 49-52: Replace “Parallel” with “Similar”, “a sequence divergence pattern” with “a pattern of sequence divergence”

Line 61: replace “one of the smallest genome sizes” with “some of the smallest genomes”

Line 62: Rewrite this sentence. It gives the impression that the tremendous phenotypic diversity of birds emerged “since the era of cytogenetics”.

Line 89: suggest replacing “retard” with “limit”

Line 112: suggest rewriting this sentence to read, “with all the cutting-edge technologies mentioned above. We corroborated our reference genome through comparisons to previously published …”

Line 113: don’t need to capitalize “Fluorescence”

Line 115: suggest removing “(chicken and turkey etc.)”.

Line 119: It isn’t clear what “they” refers to here. Is it the duck sex chromosomes, the duck, emu and chicken sex chromosomes, all three genomes together?

Line 119-121: The chronological order referred to in this sentence isn’t clear. You previously referred to the divergence time of *Anseriformes* from *Galliformes* but didn’t provide the divergence time of emu. If emu isn’t part of that chronology, then it isn’t clear because you just stated in the previous sentence that duck sex chromosomes are intermediate between chicken and emu.

Line 133: replaces “sequences” with “bases”

Line 134-137:

1) Replace “-fold” with “-X genome coverage”,

2) The Hi-C and Bionano data was from a male duck, the PacBio and 10X data from a female duck. The relatedness or not of the two sequenced individuals should be included.

3) The read N50 for the PacBio reads given in the text is 14.3 kb, but Suppl. Fig. S1 has a read N50 of 15,333 bp. The caption for Supplementary Figure S1 states that the figure is the length distribution of subreads from one SMRT cell, but there must have been multiple SMRT cells used to get 143-X genome coverage. The number of SMRT cells used should be provided either in the main text of the paper or in the caption of Supplementary Figure S1. Something like “Length distribution from one representative SMRT cell out of X SMRT cells” in the figure title for example.

Line 137: Was the illumina data generated from the same male individual as was used for the Hi-C and Bionano data? If not, how was he related or not to the other ducks used?

Line 139: need citation, preferably URL or bioproject number at the NCBI’s SRA, for the “previously published female reads”

Line 141-142: Should refer to Table 1 here since that is where this data is presented.

Line 149: what software was used for correcting the orientation errors?

Line 154: I think you need to present some data (a supplementary figure) to show that the final polished assembly is consistent with the FISH linkage map.

Line 155-156: I think this sentence is interesting, but doesn’t belong in this section of the paper. It should go where the “see below” indicates.

Line 160-162: Should state what part of the pipeline “purge haplotigs” is in.

Line 162: Change “macrochromosomes” to “assembled macrochromosomes”.

Line 164: Data should be presented to support the assembly of the microchromosomes.

Line 176: Should clarify “evolutionarily young”. Young relative to what?

Line 184: Replace “recovered” with “identified” or “annotated”, “from” with “in”, “of which” with “including”.

**Analyses**

Line 193: replace “micro-chromosome” with “microchromosome”

Line 201: rewrite to “genes on chrZ are expressed at twice the level in males versus females”

**Line 202-204: If the expression of genes on chrZ is double in males versus females, doesn’t that mean that chrZ exhibits dosage compensation in females? Maybe need to rewrite this sentence and the previous sentence to clarify.**

Line 225: delete “on”

Line 241: replace “have not found” with “did not find”

Line 247: remove “identified”

Line 251: Some more information about the location of this gene, gene annotation information or at least chromosome location, should be provided.

Line 313: suggest replacing “some tissue” with “certain tissues”.

Line 320-321: Rewrite to clarify that “their” is the sex chromosomes of Pekin duck, not Pekin duck.

Line 329: remove “of”

Line 328-350: this entire paragraph needs to be reorganized for clarity and should be broken into at least two paragraphs. One should summarize the assembly status of chrZ in the new assembly. How much sequence could be anchored into the largest scaffold? What percentage of the expected chromosome length is that? The second paragraph should be about the PAR. The next paragraph should be about the large tandem arrays. Next the duck chrW should be its own paragraph.

Line 353: “reshuffling” not “reshufflings”

Line 365: “did not find” not “have not found”

Line 374: “remove “the”

Line 378: “concentrated in those families” not “at those families”

**Line 431: “the early stage of avian sex chromosome evolution”. This is inaccurate. The emu sex chromosomes have been evolving just as long as the chicken and duck sex chromosomes. The emu sex chromosomes are just not as differentiated.**

Line 433: rewrite to “sex-linked palindromes are a feature of strongly differentiated sex chromosomes which have accumulated abundant TEs”, see above suggestion.

Line 445: SMRT cells weren’t generated, they were sequenced.

Line 614,615: replace “micro-chromosome” with “microchromosome”

Line 641: Evolutionary, not Evolution

Line 655: Table 1 and the Figure Legends should be with the figures, not with the works cited list between Table 1 and the figures.

Figure 1: This figure indicates many softwares that weren’t cited previously but should be cited.

Supplementary Figure S1: The read N50 for the PacBio reads given in the text is 14.3 kb, but Suppl. Fig. S1 has a read N50 of 15,333 bp. The caption for Supplementary Figure S1 states that the figure is the length distribution of subreads from one SMRT cell, but there must have been multiple SMRT cells used to get 143-X genome coverage. The number of SMRT cells used should be provided either in the main text of the paper or in the caption of Supplementary Figure S1. Something like “Length distribution from one representative SMRT cell out of X SMRT cells” in the figure title for example. **Could also remove this figure entirely.**

Supplementary Figure S2: Suggest changing figure title to “A representative case of assembly error correction”.

Supplementary Figure S14: Should cite ggplot2 package here.

Supplementary Figure S23: Should indicate how the strata were identified. Just a brief indication to help the reader find it in the methods.

Supplementary Table S1: Were the 10X reads paired or not? There should be an additional column in this table indicating which individuals were used for sequencing for each technology. For example, was the same female used for PacBio and 10X sequencing? Were the Illumina reads, male and female, paired-end? I think the data category for the “Read Length/N50” column should be included in each cell. Otherwise, a reasonable reader could think that the Bionano data has a read length of 325,300 basepairs (which is actually map data, not sequence reads); I suggest changing the PacBio and Bionano columns to include “(N50)” next to the basepair unit.

Supplementary Table S2: This table is very helpful to show the various parameters used in each assembly, but it also raises a couple of questions about the PacBio data. Suppl. Figure S1 showed the read length distribution from an RSII SMRT cell, but this table indicates that both sequel and RSII data was generated. It should be stated in the main text how many SMRT cells were used, and probably in Supplementary Table S1 how much sequence was generated with each type of instrument.

Line 142 – 144: Need software used for error-correction, and orientation and “connected”. I think “connected” should be replaced with “scaffolded”.

Line 145-47: What software was used for incorporating the linkage map? Suggest rewriting to: “… we incorprorated an RH linkage map[32], which reduced …”

Supplementary Table S3: What is the “ZJU1.0” column indicating here? Is it the length of the chromosome in the final polished assembly? If so, suggest changing the column name to be “chr. length” and changing the table name to be “Chromosome anchoring in ZJU1.0 assembly”.
